# Nano-Engineered Cargo for Optimizing Oral Absorption of Tizanidine Nanostructured Lipid Carriers

**DOI:** 10.34172/apb.025.45650

**Published:** 2025-10-11

**Authors:** Mohammed A. Bazuhair, Maha H. Jamal, Rawabi A. Alashari, Shakeel Ahmad, Muhammad Junaid, Maimoona Yasinzai, Muhammad Asif Nawaz, Mohannad A. Alzain, Gul Shahnaz, Ibrahim M. Ibrahim

**Affiliations:** ^1^Department of Clinical Pharmacology, Faculty of Medicine, King Abdulaziz University, Jeddah, Saudi Arabia; ^2^Centre of Research Excellence for Drug Research and Pharmaceutical Industries, King Abdulaziz University, Jeddah, Saudi Arabia; ^3^Department of Pharmacy, Quaid I Azam University, Islamabad, Pakistan; ^4^Sulaiman Bin Abdullah Aba Al-Khail-Centre for Interdisciplinary Research in Basic Sciences, Faculty of Basic and Applied Sciences, International Islamic University, H-10, Islamabad, Pakistan; ^5^Additional Secretary, Health Department, Government of Gilgit Baltistan, Pakistan; ^6^Department of Family Medicine, Faculty of Medicine, King Abdulaziz University, Jeddah, Saudi Arabia; ^7^Family Medicine and Chronic Diseases Research Unit, King Fahd Medical Research Center, King Abdulaziz University, Jeddah, Saudi Arabia

**Keywords:** Oral bioavailability, Nanostructured lipid carrier, Pharmacokinetic study, Tizanidine

## Abstract

**Purpose:**

Tizanidine (TNZ) is a muscle relaxant that works by blocking presynaptic neurons. Due to its inadequate solubility and low oral bioavailability, this medication is classified as a Biopharmaceutics Classification System (BCS) class II drug. The objective of this study was to improve the absorption of TNZ using nanostructured lipid carrier (NLCs) as a method of delivering the medicine.

**Methods:**

To achieve this objective, NLCs were synthesized using microemulsion techniques. The optimization process was conducted using Design Expert version 12 Box Behnken model. The parameters of interest were mean particle size (PS), zeta potential (ZP), and percent entrapment efficiency (EE%). The concentrations of the medication, lipid, and surfactant were varied during the optimization process. Further characterization included Fourier transform infrared spectroscopy (FTIR) and powdered X-ray diffraction (PXRD). The optimized formulation was subsequently tested for in-vitro release under varying pH conditions. The pharmacokinetic study was elicited to assess the oral bioavailability of the TNZ-NLCs in comparison to its suspension.

**Results:**

The formulation was tuned to have PS of 208 nm, a polydispersity index (PDI) of 0.221, a ZP of -18.6 mV, and an EE% of 93%. The optimized formulation remained physically stable for 12 weeks under various temperatures. The pharmacokinetic study indicated a 21-fold enhancement in AUC due to entrapment of TNZ into NLCs thereby, aligning with the aim to improve bioavailability.

**Conclusion:**

It was inferred that the inclusion of TNZ within NLCs results in its controlled release with enhanced bioavailability.

## Introduction

 Spasticity is a widely recognized condition that typically occurs following events such as stroke, multiple sclerosis, spinal cord injury (SCI), traumatic brain injuries, and other CNS abnormalities. A significant number of individuals with a spinal lesion experience a spastic movement disorder characterized by reduced speed in walking and voluntary limb motions.^1^ Epidemiologically, spasticity is a frequent occurrence following a stroke, affecting anywhere from 30% to 80% of those who have experienced a stroke. According to reports, the occurrence of spasticity in individuals with paralysis is 27% after 1 month, 28% after 3 months, 23% and 43% after 6 months, and 34% after 18 months following a stroke.^2^ Large-scale investigations regarding the natural progression of spasticity and the development of contractures are lacking. However, it has been documented that irreversible reduction in joint mobility can occur within a period of 3 to 6 weeks following a stroke.^2^

 The market has seen many medications to manage or even treat spasticity including but not limited to baclofen, diazepam, clonazepam, dantrolene and tizanidine (TNZ),^3^ of them the most common is TNZ based on the safety and efficacy,^4^ and it was for this reason that the drug was chosen. However, TNZ suffers from its fair share of disadvantages, one of which is reduced bioavailability. TNZ is a pharmacological drug that falls under the category of centrally acting imidazole derivatives. It is extensively used in clinical practice to treat both acute and chronic muscular spasms. The precise mechanism of action of TNZ is yet unclear. The drug’s alpha-adrenergic agonist mechanism of action suggests that it has the ability to regulate pain responses in the spinal cord. The motor neuron activity is reduced as a result of the activation of alpha-adrenergic receptors.^5^

 TNZ belongs to Class II of the Biopharmaceutics Classification System (BCS) and is known for its low solubility albeit with good permeability. It has a bioavailability of only 21%, meaning that 79% of the drug is lost due to metabolism and excretion prior to the onset of action.^6^ Of myriad strategies, a significant one includes stacking the active ingredient, suffering from lesser than satisfactory bioavailability, in the second-generation liposomes i.e. the nanostructured lipid carriers (NLCs).^7^ Owing to the way they are structured, the NLCs can protect the drug from the overtly acidic or basic gastrointestinal milieu. In addition, they offer an additional barrier against hepatic enzymes, thereby improving drug’s half-life and bioavailability. Moreover, the dissolution can also be improved, which contributed to the miniaturization of active ingredient’s size. Withal, mean residence time (MRT) of the drug could also improve via the pharmacokinetic meliorations. These facts serve to promote added advantages and consequences resulting in improved therapeutic efficacy, lowered toxicity, and controlled release and therapeutic index of the drug.^8^

 The manuscript is based on studies that address the effectiveness of NLCs in ameliorating the problem of TNZ associated with low bioavailability, and how the NLCs themselves fare against the *in-vitro* criteria. The studies presented point towards the fact that TNZ-NLCs can be marketed as an effective treatment modality directed towards challenges pertaining to spasticity.

## Materials and Methods

###  Materials

 TNZ was procured from Vision Pharmaceuticals (Islamabad, Pakistan).Compritol^®^ 888 ATO, Oleic acid, Tween 80, sodium chloride (NaCl), sodium hydroxide (NaOH), and Hydrochloric acid (HCl) were procured from Sigma Aldrich (St. louis, MO, USA). Potassium dihydrogen phosphate was procured from BDH Laboratory Supplies (England). Disodium hydrogen phosphate was procured from Duksan (Ansan, Korea). The remaining chemicals were all of pure analytical grade.

 High shear Homogenizer (D-91126, Heidolph, Germany), zeta sizer nano ZS90 (Malvern instruments, Worcestershire, UK), UV-visible spectrophotometer (HALO DB-20. UV-VIS double beam spectrophotometer), centrifuge (Hermle labortechnik, Z 216 MK, Germany), shaker water bath (SW22, Julabo, Allentown, PA, USA), A dialysis type cellulose membrane with a molecular weight cut-off value ranging between 12 kDa and 14kDa. Fourier-transform infrared spectrometer (FT-IR Alpha Bruker ATR eco ZnS spectrophotometer, Germany), X-Ray diffraction apparatus (BrukerAxs, D8 Advance, Karlsruhe, Germany), water distillation apparatus (Irmeco, GmbH IM50 Germany).

 Male Sprague Dawley (MSD) rats were acquired from the NIH, Pakistan and utilized in an *in-vivo* pharmacokinetic investigation. The animals were accommodated in a designated animal facility, where they received regular meals and had unrestricted access to drinking water. Furthermore, a temperature range of 24-25°C and a relative humidity of 50-60% were upheld. The approach employed for conducting animal research was adapted from the policy sanctioned by the Bioethics committee of the institute. Following the approval by the institutional committee, the studies were conducted.

###  Methods

####  Screening of solid lipids for NLCs preparation

 It was a challenge to conceive what criteria are to be used for selection of a suitable lipid, especially the solid lipid for the system. Keeping that in mind, one of the most feasible methods was to ascertain the solubility of the drug in the solid lipid as it maintains the structural integrity of the NPs and the liquid lipid within. It was necessary to compare various lipids, including Gelucire 44/14, stearic acid, Precirol^®^ ATO5 (PRE), and Compritol^®^ 888 ATO (COM). The procedure enacted such that 5 g of the aforementioned lipids were melted and kept at temperatures 5-10 degrees higher than the highest melting point i.e. 80 °C. The operation conditions included agitation for 24h at 100 rpm. Afterwards, 100 or 200 mg of drug was added to cognize the saturation solubility. The solubility of TNZ in the respective lipids was determined via visual or organoleptic assessment and the insolubility was concluded following the emergence of precipitated drug, post settling, or insolubility. The method was in agreement with the one mentioned in the literature.^9^ However, for liquid lipid we used oleic acid only, to take advantage of its anti-inflammatory properties.^10^

####  Preparation of TNZ-NLCs

 TNZ-NLCs were synthesized using a modified microemulsion technique. Alternative methods for producing NLCs do exist, but they typically require specific tailored equipment, suffer from formulation stability issues, or involve the use of potentially toxic organic solvents. Considering these drawbacks, microemulsion was selected as the optimal choice due to its efficiency, simplicity, low energy requirements, and suitability for laboratory settings. The approach described in the literature was used to successfully construct NLCs of TNZ.^11^ In order to achieve a homogeneous and transparent blend, the solid lipid, COM, and the liquid lipid, oleic acid, were heated to a temperature of 80 °C (Figure S1). This temperature was chosen to be somewhat higher by 5 to 10°C than the melting point of the solid lipid employed in this particular experiment. The aqueous phase was created by combining the medicines, surfactants, and distilled water, which were then heated to the desired temperature using a hot plate stirrer. At a rotational speed of 750 revolutions per minute (rpm), the aqueous phase was added to the molten solid lipid. The temperature was then kept at 80 °C while swirling the mixture continuously. The pre-emulsion obtained was subjected to homogenization for a duration of 15 minutes at a speed of 10,000 rpm in a high-shear homogenizer. To obtain TNZ-NLCs dispersion, one volume of the micro emulsion was combined with 9 mL of distilled water that had been chilled to 4 °C.^11^

####  Optimization of TNZ-NLCs

 The optimization of TNZ-NLCs was conducted using the Box-Behnken model (3 factors, 3 level) in Design Expert software version 12. The ratios of solid lipid (COM), drug (TNZ), and surfactant (Tween 80) were altered, and the impact on particle size (PS), zeta potential (ZP), and percentage of encapsulated drug (% EE) were assessed.

####  Particle size, polydispersity, and zeta potential analysis

 The mean PS, polydispersity index (PDI), and ZP of the TNZ-NLCs dispersion were measured using a ZS 90 Zetasizer that was equipped with a 635 nm He-Ne laser. The measurements were conducted using the Zetasizer software v 6.34, with fixed light incidence angles of 90° and 25° (Malvern instruments Ltd, UK). Before doing the analysis, 10 µL of the sample was mixed with 1 mL of deionized water and vigorously mixed for 1 minute.

####  Entrapment efficiency of TNZ loaded NLCs

 The concentration of the medication that was not bound to other substances in the liquid above was utilized to determine the effectiveness of trapping TNZ-NLCs. The formulation was subjected to centrifugation at 20,000g for 1.5h at a temperature of 4°C. The transparent liquid obtained was diluted in distilled water at a ratio of 1:10 (1 mL of liquid in 10 mL of distilled water). Subsequently, the quantity of unbound medication was assessed by employing a UV visible spectrophotometer configured to measure at 235 nm for TNZ. The entrapment efficiency (EE%) of the TNZ -loaded NLCs was calculated using the following formula:

 % Entrapment efficiency = Wt – Wf / Wt × 100

 Where, Wt is the total drug concentration

 And Wf is the concentration of free drug in supernatant of NLCs dispersion.

####  Fourier transformed infrared spectroscopy (FTIR)

 This experiment utilized FTIR spectroscopy to investigate potential chemical interactions between the drug TNZ and excipients such as lipids (COM and oleic acid) and the surfactant Tween 80. The specific instrument utilized was the Bruker Alpha II with opus software version 8.0. The samples were scanned using a wavenumber range of 400 to 4000 cm^-1^ and the results were plotted using Origin Pro 2022 software.^12^

####  X-ray diffraction (XRD)

 The XRD analysis was conducted to cognize the polymorphic nature of TNZ, COM, and TNZ-NLCs. Briefly, Cu K radiation was utilized, with a current of 40 mA and a constant voltage of 40 kV. Scanning was performed within a 2θ range of 5° to 80°, with a gradual increase of 5°/min.

####  In-vitro release profile

 The study on the release of optimized TNZ-loaded NLCs was conducted at pH levels of 1.2, 6.8, and 7.4. A dosage of 5 mg of TNZ was utilized in each both the TNZ solution and the TNZ-loaded NLCs. The technique employed was the dialysis bag method. The respective dialysis bags (with 14 kDa molecular weight cut off) were filled with a TNZ-drug solution and TNZ-loaded NLCs, respectively, holding TNZ equivalent to 5 mg. The bag’s extremities were thereafter secured with thread and immersed in beakers filled with 500 mL of dissolving media. There were six beakers used in the experiment. The first beaker contained TNZ-loaded NLCs at pH 1.2, the second beaker contained TNZ drug solution at pH 1.2, the third beaker contained TNZ-loaded NLCs at pH 6.8, the fourth beaker contained TNZ drug solution at pH 6.8, the fifth beaker contained TNZ-loaded NLCs at pH 7.4, and the sixth beaker contained TNZ drug solution at pH 7.4. These beakers were placed in a shaking water bath with a temperature of 37 °C and a rotational speed of 80 rpm. At regular time intervals (such as at every 0.25 h, 0.5 h, 1 h, 2 h, 4 h, 6 h, 12 h and 24 h). In order to maintain consistent sink conditions, a volume equivalent to 1 mL of the respective media (NLCs dispersion and Drug dispersion) was aliquoted from each beaker and substituted with an equivalent amount of respective buffer solution. Each aliquot was then analysed for the quantity of TNZ contained wherein, the absorbance of each sample was measured at a wavelength of 235 nm using a UV spectrophotometer. This allowed for the quantification of the drug content in each sample by comparing the obtained data to a standard curve.

####  Drug release models

 Various models that conceive the release mechanism for the system were applied. Of them the notable ones were zero order, 1^st^ order, Higuchi, Korsmeyer-Peppas and Hixon-Crowell models. The model exhibiting the highest R^2^ had the strongest argument to be unanimously selected as the one explaining the release of the drug.^13^

####  HPLC Method for the determination of TNZ in plasma

 To determine the concentration of TNZ in the blood samples it was necessary to develop a method for the quantitation in an HPLC.

####  Validation of the method

 The validation of TNZ by HPLC was performed considering several critical parameters. The mobile phase, maintained at a flow rate of 1.0 mL/min, ensured a continuous and stable elution, which is essential for accurate separation and identification of TNZ. The mobile phase consisted of a 1:1 (v/v) mixture of acetonitrile and an aqueous component, typically water or buffer, to facilitate efficient elution and separation of TNZ from the plasma matrix. A fixed injection volume of 20 µL was used to maintain consistency and allow precise quantification of the analyte. Chromatographic separation was achieved using an Inertsil ODS-4 C18 column (4.6 × 150 mm, 5 µm particle size), selected for its compatibility with TNZ and its capability to deliver effective resolution. The column temperature was maintained at 25 °C throughout the analysis to ensure reproducibility and stable retention times.

####  Linearity and calibration curve for TNZ 

 The TNZ stock solution (1,000 µg/mL) was made by precisely measuring 50 mL volumetric flask and filling it to the desired volume with the mobile phase. The solution underwent sonication for a duration of 30 minutes at ambient temperature. Working solutions (dilutions) for HPLC injections were made from the stock solution. Various dilutions bearing the concentrations of 1.5, 3.125, 6.25, 12.5, 25, 50, and 100 µg/mL were prepared so that an accurate calibration curve could be deployed. The mobile phase used for the injections consisted of a mixture of 0.5 mM phosphate buffer and acetonitrile in a ratio of 40:60 (v/v). Additionally, an appropriate amount of plasma was added to the mobile phase. The C18 column bearing the dimensions above was employed. The UV detector was set to a wavelength of 230 nm, and the flow rate predetermined. Prior to injection, solutions were passed through a 0.45 mm membrane filter for filtration. The solution underwent sonication and vortexing, followed by precipitation of plasma proteins using 1200 µL of cold methanol and acetonitrile in a 1:1 ratio.^14^

####  In-vivo pharmacokinetic studies

 The study utilized male albino rats weighing between 240-250 g, which were sourced from the NIH, Islamabad, Pakistan. Ethical permission was obtained from the research institution, and the protocol was approved by the aforementioned organization’s ethical committee. The animals were kept at the room temperature and had a sleep/wake cycle of 12 hours. Prior to administering the TNZ solution and the TNZ-loaded NLCs formulation, the animals underwent a 12-hour fasting period with unrestricted access to water. The animals were allocated randomly into two groups, each consisting of five rats. The TNZ dispersion and TNZ-loaded NLCs were administered orally to rats at a dosage of 3 mg/kg for TNZ. The formulations were administered via oral gavage, delivering the dose directly to the lower part of the rat’s oesophagus. The doses of oral formulations, specifically TNZ-loaded NLCs, were prepared by dispersing NLCs containing TNZ equivalent to 3 mg, in an aqueous phase consisting of Tween 80 (1% w/v) and distilled water. Alternatively, the solution was prepared by dissolving 3 mg of TNZ in an aqueous phase consisting of distilled water and Tween 80 (1% w/v), resulting in a final volume of 20 mL. Prior to administration, the solutions underwent sterile filtration using a 0.45 µ syringe filter. Blood samples (0.2 mL) were obtained from each rat by conducting venipuncture on the tail vein and collecting them into EDTA-coated tubes as reported by Zou et al.^15^ Orally dosed rats were sampled at 0.5, 1, 2, 4, 6, 12, 24 and 48 h. post drug treatment. The plasma was promptly collected by centrifugation at a force of 3600 g for a duration of 10 minutes at a temperature of 4 °C. Subsequently, it was stored at a temperature of -20 °C for future analysis. Specimens of venous blood were obtained and placed into tubes containing EDTA.^16,17^ The specimens were centrifuged at 5000 rpm for 15 minutes to separate plasma from blood cells. For the extraction of both drugs from the blood samples, 200 µL of each sample was mixed with 200 µL of ethyl acetate, followed by vortexing for 3 minutes and centrifugation at 1000 rpm for 10 minutes. Plasma was obtained by adding a chilled mixture of methanol and acetonitrile (1:1, v/v), which was then subjected to centrifugation to remove protein residues. The resulting supernatant was collected and filtered through a 0.45 µm syringe filter prior to injection into the high-performance liquid chromatography (HPLC) system. Additionally, the solution was sonicated or vortexed, and plasma proteins were precipitated using 1200 µL of cold methanol and acetonitrile in a 1:1 ratio.^18^ Following this, the pharmacokinetic parameters maximum concentration (C_max_), peak time (t_max_), area under the curve (AUC_0-t_, AUC_0-∞_), mean resident time (MRT), AUMC (Area under the moment curve) and half-life (t_1/2_) were determined using non-compartmental analysis with the PK solver software.^19^

###  Statistical analysis

 All the purported results were statistically ascertained via student t-test or ANOVA with post hoc Tukey analyses, where applicable, using GraphPad Prism v 9.0.121. The criteria for the significance were *P* value wherein, *P* < 0.05 was seen as a measure of established significance and whereas, the lesser *P* value only strengthened the significance. The results of zeta analyses were presented as mean ± SD whilst the calculation was conducted in Microsoft excel.

## Results and Discussion

###  Screening of solid lipids for SLN development

 As contemplated upon it, the solubility of TNZ in various solid lipids was evaluated so that the most feasible lipoidal system could be confirmed. By the assessment of the solubility in lipid it was found that 100 mg of the TNZ failed to dissolve in stearic acid and Gelucire 44/14 whereas it did dissolve in both COM and PRE. In the second step at 200 mg, COM individually was the molten liquid that adequately dissolved TNZ whereas the same could not be observed for the PRE as visible insolubility could indicate that. The importance of this miscibility study stems from the notion that higher solubility of the drug is paramount to proportionate entrapment and loading capacity.^20^ COM, was selected as the lipid based on the fact that only it was able to dissolve the drug completely at a percentage of 5% w/w. Whereas, the surfactant as in line with the literature was chosen at the level of 1.5% w/w.^21^

###  Optimization of TNZ loaded NLCs

 The formulation of TNZ-loaded NLCs was optimized using the Box-Behnken design, a model in Design Expert. The impact of solid lipids (COM), liquid lipid (Oleic acid), surfactant (Tween 80), and TNZ on optimization parameters such as ZP, EE%, and PS were examined. Table 1 illustrates the impact of various independent variables on dependent variables. The optimal formulation was F11.

**Table 1:** Optimization chart amidst the dependent and independent variables whereas, the highlighted row pertains to the optimized formulation

**Std**	**Run**	**Lipid (mg)**	**Surfactant (mg)**	**Drug (mg)**	**Particle size (nm)**	**Zeta Potential (mV)**	**EE (%)**
1	13	70	70	7.5	238.0 ± 5.7	-15.9 ± 3.1	80.1 ± 2.9
2	8	90	70	7.5	325.2 ± 13.4	-7.9 ± 1.7	57.3 ± 1.2
3	14	70	100	7.5	198.9 ± 8.1	-18.7 ± 5.1	71.4 ± 1.7
4	1	90	100	7.5	275.7 ± 9.5	-11.7 ± 1.2	59.1 ± 1.8
5	11	70	85	5	207.8 ± 2.5	-18.0 ± 0.6	93.5 ± 2.0
6	3	90	85	5	255.2 ± 10.3	-12.3 ± 2.7	67.8 ± 2.6
7	6	70	85	10	235.0 ± 11.1	-16.1 ± 2.7	79.9 ± 2.1
8	7	90	85	10	318.3 ± 12.7	-8.3 ± 2.3	62.1 ± 1.6
9	9	80	70	5	249.1 ± 9.4	-13.8 ± 2.9	72.6 ± 1.8
10	12	80	100	5	211.0 ± 2.9	-16.9 ± 2.1	68.1 ± 1.1
11	2	80	70	10	295.3 ± 7.8	-10.1 ± 1.6	64.3 ± 2.9
12	10	80	100	10	259.0 ± 8.4	-13.5 ± 3.3	61.3 ± 1.5
13	4	80	85	7.5	218.8 ± 3.9	-14.1 ± 3.1	73.2 ± 1.1
14	5	80	85	7.5	219.7 ± 6.2	-14.3 ± 1.5	73.8 ± 1.3

All the values pertaining to the dependent variables are expressed as mean ± SD (n = 3).

####  Effect of variables on particle size

**Figure 1:**
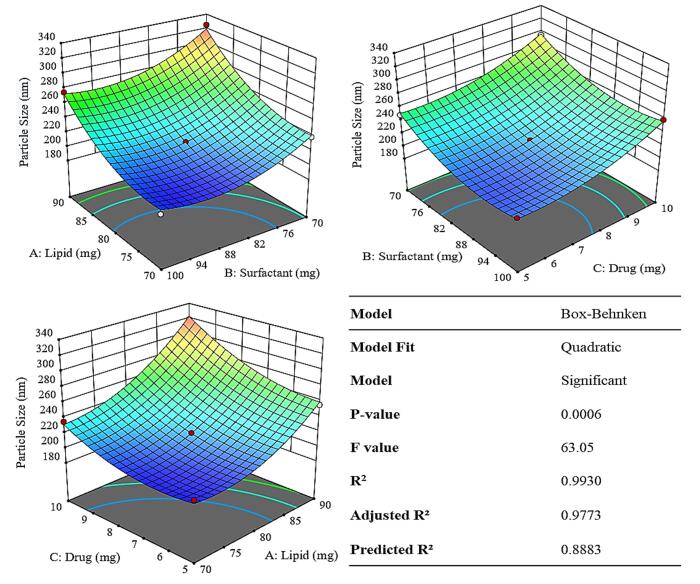
Graphical representation of parameters affecting PS

Table 1 in addition to Figure 1, indicate that elevating the concentration of solid lipid from 70 mg to 90 mg (out of 100 mg with corresponding amount of liquid lipid) increased the PS from 198.9 nm to 325 nm. Reducing solid lipid concentration decreases PS, while increasing liquid lipid concentration, due to less solid lipid, lowers viscosity and interfacial tension. Similar findings were noted by Sanad et al, wherein, the PS reduced proportionally with low drug concentration and vice versa for high concentration Literature states that higher molten lipid viscosity hinders phase dispersion, increasing PS as oxybenzone NLC drug concentration rises.^22^

 Furthermore, as shown in Figure 1, the concentration of Tween 80 was in hyperbolic relationship to the reduction in PS. Initially, the relationship between the surfactant and particle size followed inverse pattern wherein, elevating the tween 80 concentration form 70 mg to 85 the PS reduced from 325 nm to 218 nm. Following this as exhibited in Table 1, the pattern stayed consistent until it followed a positive linear pattern where the PS amplified to 275 nm for 100 mg of surfactant. Literature indicates that PS grows with higher solid lipid concentration and shrinks with more surfactant. This is because higher solid lipid concentration, due to reduced liquid lipids, enhances mechanical strength, increasing interfacial tension and viscosity, thus enlarging PS. More surfactant lowers surface and interfacial tension, forming smaller particles.^23^

 Moreover, augmenting the concentration of TNZ from 5 to 10 mg equivalently improved the PS thereby, exhibiting a direct relation as evidenced in Figure S2. The reason for that is, as drug entrapment efficiency increases, nanoparticle size tends to rise due to the additional space needed to accommodate drug molecules within the polymer matrix.^24^

####  Effect of variables on zeta potential 

**Figure 2:**
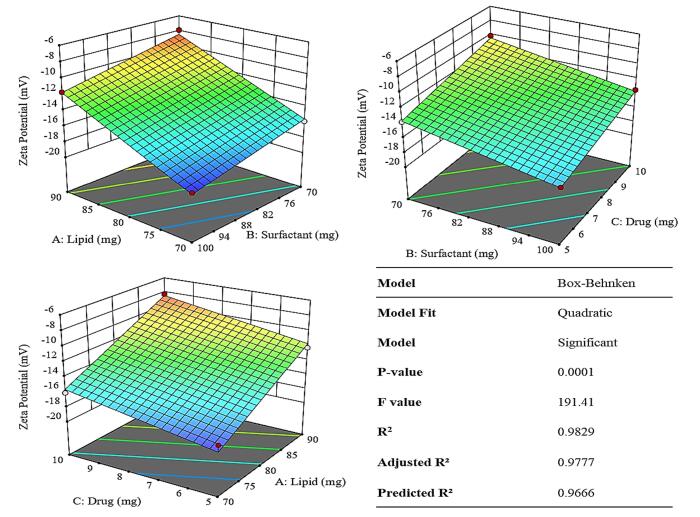
Plots explaining the impact of independent variables on the ZP

 Increasing the concentration of COM from 70 mg to 90 mg and consequently increasing liquid lipid tended to increase the ZP, albeit in small capacity from – 7.9 mV to – 18.7 mV as is elicited by Figure 2 and supported by Figure S3. Literature suggests that the increase in Compritol concentration in nanoparticles increases the surface charge (zeta potential) primarily because Compritol alters the lipid matrix and surface chemistry of the nanoparticles. As more Compritol (a solid lipid mainly composed of glycerol behenate) was incorporated, it changed the distribution of charged groups and the electrical double layer at the particle surface, which leads to a higher measured ZP.^25^

 As was substantiated in Table 1, raising the concentration of the surfactant (Tween 80) lead to a reduction in the ZP value, from – 7.9 to – 18.7 mV. Even though, tween 80 itself is non-ionic the decrease in ZP could be attributed to increase in surface area via size reduction.^26^ Figure 2 also illustrated that the ZP exhibited an inverse relationship with an increase in the concentration of the TNZ. It was due to elevation in PS wherein the surface area decreases.^27^

####  Effect of variables on % entrapment efficiency

**Figure 3:**
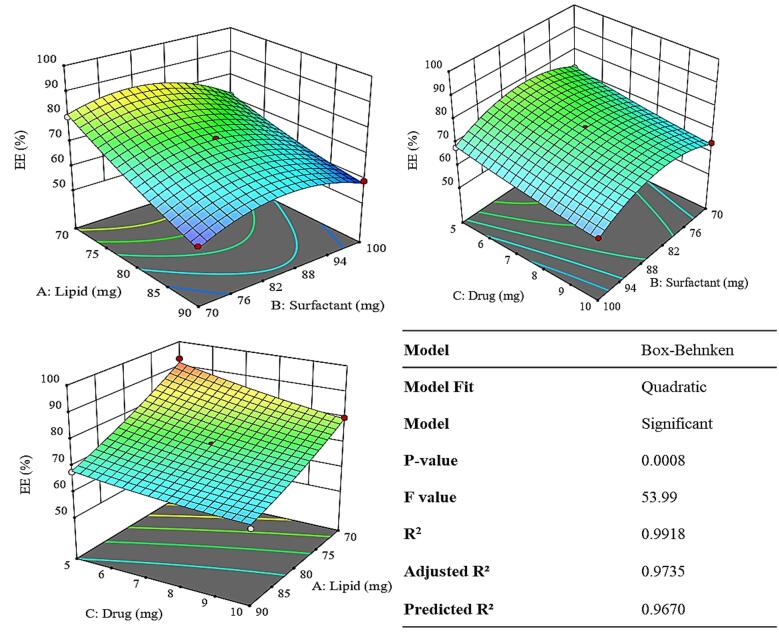
Plots explaining the impact of independent variables on the EE%

 As the solid lipid content increases from 70 to 90 mg there was a corresponding drop in the EE% i.e. from 93 to 57 % as exhibited in Table 1. The reason could be due the fact that, the change in the solid-to-liquid lipid ratio influence drug entrapment and leakage. A higher solid lipid fraction can make the lipid matrix more crystalline and rigid, thereby squeezing out the drug or causing phase separation, resulting in leakage.^28^ Figure 3 shows that by augmenting the concentration of the surfactant, the percentage of entrapment initially rises 85 mg and subsequently declines when the concentration of surfactant crosses (Figure S4). It could be due the fact that when the concentration of surfactant thresholds a certain limit i.e. the CMC or the plateau effect the size does not correspondingly respond to the surfactant effect. Post this limit the surfactant may even behave inversely.^29^ Higher drug concentrations lead to a decrease in entrapment efficiency from 93 to 61.3 % as elicited in Table 1. There could be two reasons for this the first being the drug may saturate the nanoparticle matrix capacity, leading to inefficient encapsulation. Whereas it was also plausible that excess drug can precipitate outside the nanoparticles or remain unentrapped, reducing the percentage encapsulated.^30^

###  Particle size, PDI and zeta potential of optimized TNZ loaded NLCs formulation

**Figure 4:**
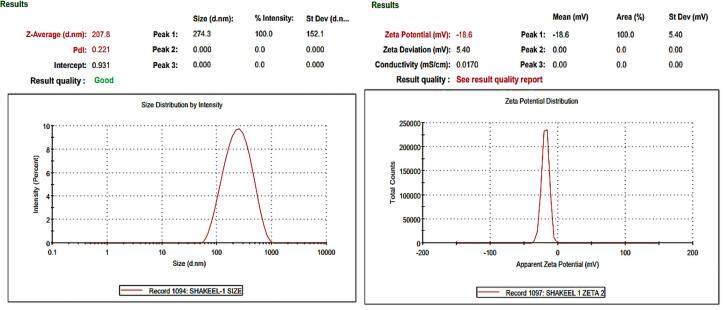
The figure illustrates the mean PS (nm) at left and Zeta potential (mV) of optimized TNZ-NLCs at right

 The optimised TNZ-loaded NLCs exhibited a mean PS of 207.8 ± 2.5 nm, a ZP of -18.0 ± 0.6 mV, and a PDI of 0.221 ± 0.005 as exhibited in Figure 4. These results indicate that the particles were in nano-size range and were monodispersed, as the PDI was less than 0.5. The PS of less than 250 nm shows that the Optimized NLCs are ideal candidate to improve the bioavailability of TNZ whereas, PDI indicates homogeneity in mean PS, whilst the ZP values hint at the stability of NLCs across the shelf life.^31^

###  Entrapment efficiency of optimized TNZ-loaded NLCs formulation

 The entrapment efficiency of the optimized TNZ loaded NLCs for TNZ was determined to be 93%. The marginal effect of lipids as an independent variable on EE% can be explained by decreased interfacial tension between the lipid phase and the drug, increasing drug encapsulation efficiency as surfactant concentration rises. This aligns with previous findings.^32^ TNZ’s entrapment efficiency decreases after an inflection point, typical of quadratic models, as drug concentration increases. This was due to limited lipid molecules available to absorb higher drug concentrations, as noted in the literature.^33^ Higher medication and lipid concentrations increase EE%, with more lipid content enhancing drug encapsulation by reducing its partition in the outer phase.^33^

###  FTIR analysis of TNZ-NLCs

**Figure 5:**
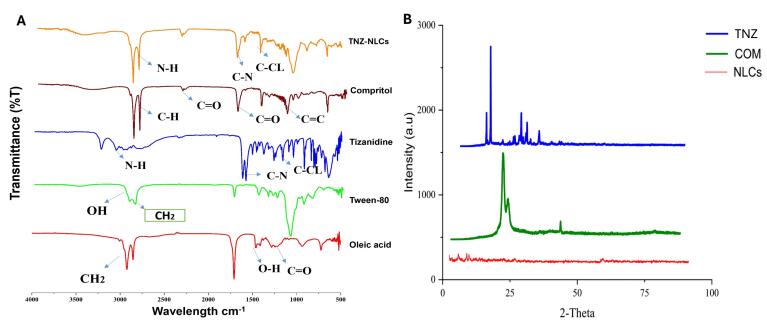
Solid state characterization like (A) Fourier Transform Infrared Spectroscopy (FTIR) and (B) Powdered Xray Diffractometry (PXRD) exhibiting chemical interaction amidst the components of formulation, and the polymorphic nature of the components and formulation, in a respective manner

 The purpose was primarily to examine the chemical interactions between the medicine and its excipients, such as lipids and surfactants. The FTIR range utilized ranged from 500 to 4000 cm^-1^. Figure 5 displays the FTIR spectra of TNZ-loaded NLCs. The peaks ranging from 3200 to 3400 cm^-1^ corresponded to the N–H stretching vibration of the amino groups. The vibrations observed at 1750-1735 cm^-1^ correspond to the (C = O) bond of the carbonyl group, while the vibrations observed at 1690-1640 cm-1 indicate the existence of an imine group (C = N). The stretching observed at 1454 cm^-1^ corresponds to the C–N stretching vibration originating from the TNZ proline ring. The bands found at 3554 cm^-1^ correspond to the O–H stretching vibration originating from the crystallization of water. The presence and mobility of all characteristic peaks in TNZ-loaded NLCs suggest that the medications have been successfully incorporated into the lipid structure, and there was no interaction between the drug molecules and lipids at the molecular level. Our selection of the lipid and the surfactants to incorporate TNZ was based on the literature that indicated no interaction between them and drugs with similar properties.^12^

###  XRD analysis of TNZ-loaded NLCs

 Determination of the polymorphism in the formulation and its components could be construed from the evidence exhibited in Figure 5. The drug, TNZ, elicited characteristic peaks at the angles of 10.5, 12.2, 24.6, 27.09 and 32.02 degrees thereby, substantiating the arguments in favour of crystalline nature. On similar grounds, the peaks at 20.89, 22.63 and 43.67 degrees the lipid had an amount of crystallinity in it. However, similar deliberation could not be extrapolated on the NLCs which exhibited amorphous patterns of reflection. The results showed significance in explaining that the NPs were in fact amorphously encapsulating the drug within it as established in the entrapment results. Furthermore, the conversion to the amorphous form indicated the elevated solubility when the drug was released in the body. This may be explained by conversion of COM, and TNZ from crystalline to amorphous forms that did not respond to the light at 0.154 nm thereby, were devoid of exhibiting diffraction across the various facets of their surfaces.^34^

###  In-vitro release studies

*In-vitro* release study was carried out to assess the release pattern of TNZ including TNZ dispersion and TNZs at varied pH in the appropriate medium.

####  In-vitro drug release at pH 1.2

**Figure 6:**
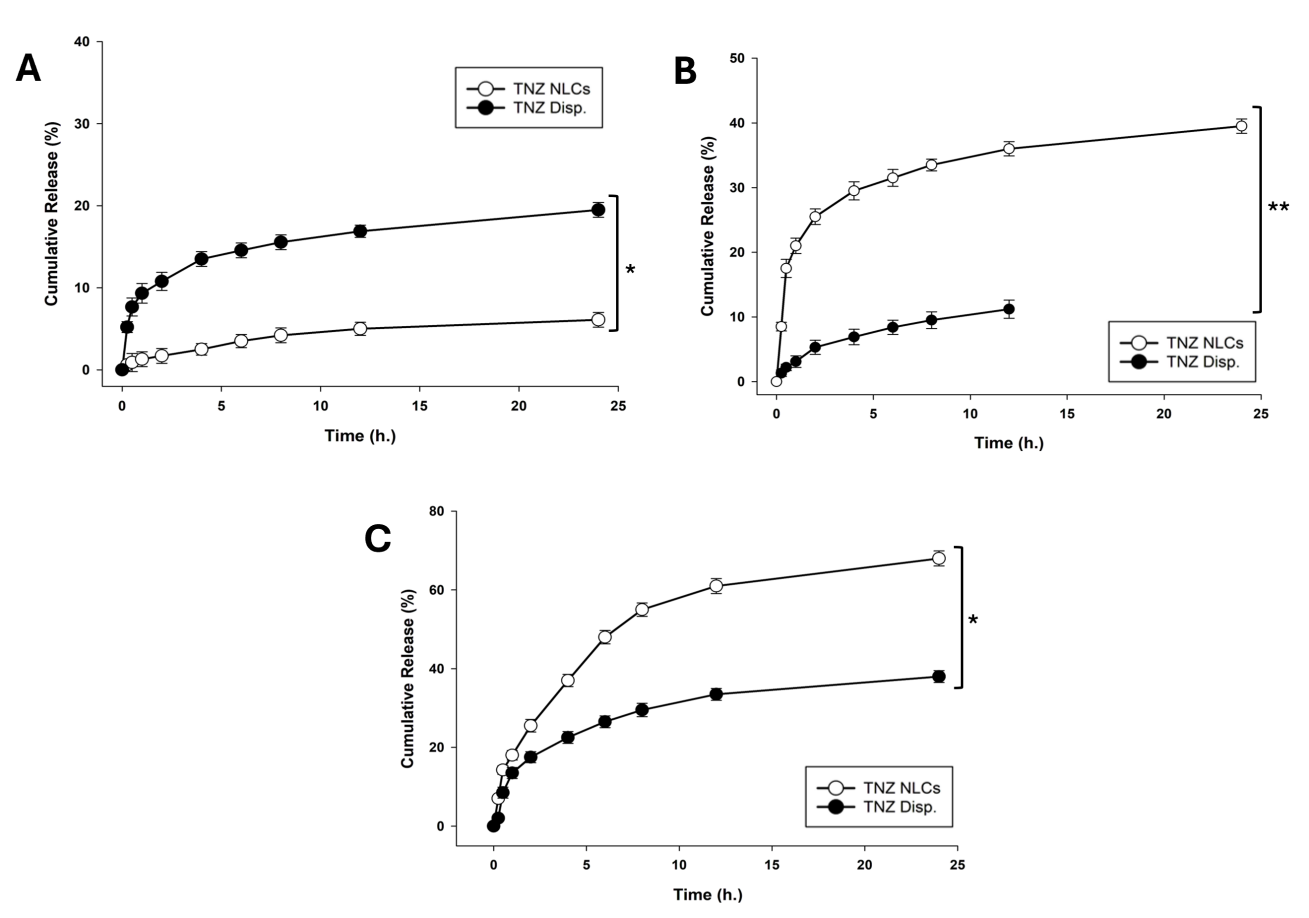
Cumulative Release profiles across the GI pH for TNZ and TNZ NLCs(A) Release profile of TNZ and TNZ NLCs at gastric pH (1.2). (B) Release profile of TNZ and TNZ NLCs at the intestinal pH (6.8). (C) Release profile for TNZ and TNZ NLCs at colon pH or blood pH (7.4) The values are expressed as mean ± SD (n = 3). Whereas ^*^P < 0.05 and ^**^ P < 0.01, represented the degree of significance compared to the TNZ dispersion

 Although the gastric retention time is less than 1 h, we explored the behaviour of the NLCs, and the drug entrapped by a 24-h study. The results plotted in Figure 6A, indicated a 20% release during the course of the inquiry with negligible burst effect, from the TNZ dispersion. On the other hand, the TNZ-NLCs constituted 6% of drug dissemination in 24 h. This difference could be explained by the nanovesicles restricting the dissolution profile of TNZ. The results at pH 1.2 also insinuated that after 6 h the release saturated, suggesting the refractiveness of the system to the pH. This was desirable considering the fact that the drug release would be ideal at the physiological pH. These results are consistent with the known pKa of glyceryl behenate. The likely cause is that the nanoparticles are covered by a uniform coating of Tween 80 which make it more stable at low pH environment that way it mimics the release of medicine, in addition to the low responsiveness of the COM layer itself.^35^ The delayed release of the drug may be attributed to the pH-dependent solubility of TNZ, which exhibits less release at acidic pH levels relative to intestinal and plasma pH levels.^35^

####  In-vitro drug release at PH 6.8

 On the other hand, as elicited by Figure 6B, only 40% of the drug release was observed at the colon pH of 6.8 in 24 h for the TNZ dispersion. Yet, NLCs exhibited an 11% release in 24 h. There was a significant difference (*P* < 0.01) amidst the NLCs and TNZ dispersion in terms of release behaviours which could be desirable as NLCs bear high absorption owing to the nanoparticulate nature in addition to the fact that, lesser release in the GIT, implies the intactness of the vesicles at the absorption site. The resultant effect would be a consequent high bioavailability.^36^ The release of 40% was in direct correlation with the pKa of the COM.^37^ Moreover, the burst release was non-discernible, suggesting high entrapment efficiency of the TNZ-NLCs.The findings were consistent with a prior published study.^38^ The decreased release of the substance at the pH level of the intestines compared to the pH level of the blood plasma may be explained by the protective effect of Tween 80 present in NLCs, which prevents their breakdown. As a result, this shielding action allows NLCs to preserve their integrity and promote their potential absorption by endocytosis during intestinal uptake. The inclusion of Tween 80 helps to the better performance of NLCs by enhancing intestinal permeability as stated in literature.^39^ However, the impact of Tween 80 on permeability remains out of the scope of this manuscript and needs to be explored.

####  In-vitro drug release at pH 7.4

 The results presented in Figure 6C, were quite the contrary, when the release study at pH 7.4 showed 69% release at physiological pH for TNZ-NLCs across the 24h. One might argue that this concentration is less than desirable, yet it must be noted that this concentration is a marked improvement in comparison to the oral bioavailability of the conventional drug formulation. Whereas TNZ flaunted 38% of release which is commensurate to the pKa value and the pH of the drug.^37^ The difference was significant (*P* < 0.01) amidst the TNZ-NLCs and TNZ dispersion. The initial release observed in the solution is attributed to its hydrophilic properties and solubility that is dependent on pH. On the other hand, in the case of NLCs, the drug diffuses gradually from the core into the release medium, resulting in a continuous release of the medication over a period of 24 h.^40^

###  Kinetic models for the determination of TNZ release mechanism

 The drug release mechanism from TNZ-loaded NLCs was assessed by applying various kinetic models to the release data. After applying the release model, the Korsmeyer-Peppas model was selected as the best fit, determined by its greatest R^2^ value (0.9918). Table S1 presents various kinetic models along with their corresponding R^2^ values. As per Table S1 the value of 0.462 is very close to the Fickian threshold but slightly higher, implying that the main drug release mechanism was diffusion through the NLC matrix, with only a minor contribution from other processes like lipid erosion or swelling. This means the drug molecules are primarily moving out of the carrier due to a concentration gradient, and the structure of the NLCs remains largely intact during the release process. The near-Fickian release is advantageous in many cases, especially for sustained drug delivery thereby, predictable and stable release kinetics.^41^

###  In-vivo pharmacokinetic study

####  HPLC validation of TNZ

 The identification of TNZ was conducted at a precise wavelength of 230 nm. The selection of this wavelength was based on the absorption characteristics of the molecule, which guarantees precise and highly sensitive detection. From the results it can be concluded that at 230 nm, the drug could be sufficiently detected with 800 mAU amplitude and discernible peak without any impurities. Moreover, the retention time (RT) could be recognized as 3.69 min for TNZ. Furthermore, the calibration curve was plotted, exhibiting an R^2^ value of 0.9991, thereby forming the basis for the calculation of plasma concentrations following rodent sampling in the pharmacokinetic analysis.

####  Pharmacokinetic parameters

**Figure 7:**
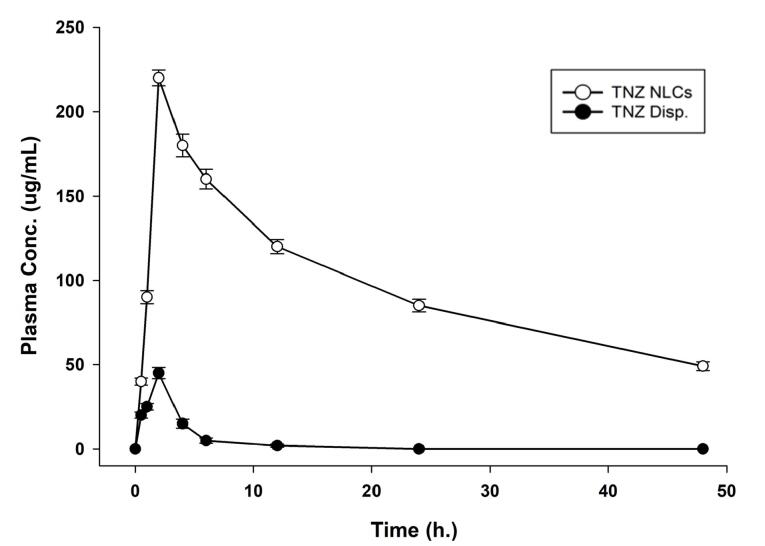
Exhibiting the comparison in the plasma concentration (PO) profile of the NLCs with the TNZ. The dots symbols corresponding to the connecting lines in the plot and error bars are indicative of Mean ± SD

 The results demonstrated that TNZ-NLCs significantly enhanced the plasma concentration of TNZ when compared to orally administered TNZ solution, as could be observed in Figure 7. As evident from the results presented in Table 2, the AUC improved significantly (*P* < 0.0001, CI 99.99%) from 186.192 ± 13.839 (TNZ) to 3980.756 ± 24.221 (TNZ-NLCs) with a 21-fold improvement owing to the NLCs bearing higher V_d_ and curtailed metabolism. On the other hand, the half-life improved by 8.34 times (*P* < 0.0001, CI 99.99%). The reason was that the NLCs offered protection against the first pass effect.^42^ The extended half-life of NLCs suggests that they allow for a slower elimination rate of the drug, potentially leading to a longer duration of its effects compared to the TNZ solution. This demonstrates a substantial enhancement in the absorption and availability of the substance in biological systems. Similarly, as supported by Figure 7, there was 4.68-fold (*P* < 0.0001, CI 99.99%) spike in the C_max_, which could be attributed to the fact that the NLCs bear higher absorption compared to the drug in its raw form. Moreover, marked improvements could be observed in other pharmacokinetic parameters including but not limited to MRT (*P* < 0.001, CI 99.9%) and AUMC (*P* < 0.001, CI 99.9%). This indicates that NLCs have gradually increased the exposure of the given medication in the bloodstream, hence broadly impacting the drug’s otherwise compromised bioavailability. It is pertinent to mention that the increase can be ascribed to various variables, including improved gastrointestinal permeability and bioavailability through the use of surfactants, absorption of nanoparticles in the gastrointestinal tract, accelerated dissolution rate, and reduced degradation.^43^ A notable limitation of the present study is the absence of comprehensive toxicity and safety evaluation of the developed TZN-loaded NLC formulations. While the current investigation focused on pharmaceutical characterization, drug release kinetics, and pharmacokinetic enhancement, future studies should incorporate detailed toxicity assessments including cytotoxicity studies using relevant cell lines, hemocompatibility evaluation, and in vivo safety studies to establish the biocompatibility profile of the lipid nanocarriers^44^ However, despite the limitation our results were in line with previous attempts to utilize NLC approach to enhance oral bioavailability of drugs.^45^

**Table 2:** Pharmacokinetic parameters for comparative analyses

**Parameters**	**Unit**	**TNZ-NLCs**	**TNZ**
Lambda_z (K_el_)	1/h	0.033 ± 0.004***	0.056 ± 0.0026
t_1/2_	h	20.45 ± 0.651****	2.45 ± 0.125
T_max_	h	2.00 ± 0.011	2.00 ± 0.029
C_max_	μg/mL	211.036 ± 9.521****	45.00 ± 2.958
C_last_obs_/C_max_		0.153 ± 0.033***	0.012 ± 0.003
AUC_ 0-t_	μg/mL*h	3980.756 ± 24.221****	186.192 ± 13.839
AUC _0-inf_obs_	μg/mL*h	4931.077 ± 43.226****	196.185 ± 23.557
AUC _0-t/0-inf_obs_		0.807 ± 0.027	0.950 ± 0.058**
AUMC _0-inf_obs_	μg/mL*h^2^	143007.767 ± 93.878****	2263.771 ± 44.644
MRT _0-inf_obs_	h	29.001 ± 3.347***	11.540 ± 1.026

All the data are presented as mean ± SD (n = 5). Whereas MRT, Mean Residence Time. Lambda Z, Terminal elimination rate constant (K_el_). T_1/2_, Half-life. T_max_, time to reach maximum concentration. C_max, _Maximum plasma concentration AUC, Area under the curve. AUMC, Area under the moment curve. C_last_obs_/C_max_, ratio of the last observed plasma concentration of drug to maximum plasma concentration a drug. *P < 0.05, ** P < 0.01, *** P < 0.001, **** P < 0.0001.

###  Stability study 

 Following the guidelines set by the International Council for Harmonization (ICH), the stability data of TNZ-NLCs was collected at two distinct temperatures, namely 25 ºC and 40 ºC, for the duration of 6 months. The findings in Table 3 suggested that there were no significant changes in the PS, PDI, and ZP of the TNZ-NLCs, indicating that the nanoparticles remained stable. All the results were presented in triplicate. The results of this study were consistent with the findings reported by Shah et al.^46^ Elsewhere, the stability study demonstrated that there were no significant differences between the initial and final values of PS, PDI, ZP, and %EE for NLCs.^47^ This lack of variation strongly supports the conclusion that TNZ-NLCs are able to maintain their stability throughout the evaluation period.

**Table 3:** Stability data for the TNZ-NLCs at ambient and accelerated conditions of temperature and relative humidity

**Parameters **	**Ambient conditions **				**Accelerated conditions **			
Storage temperature	25 °C ± 2				40 °C ± 2			
Relative humidity	60% ± 5%				75% ± 5%			
Time (months)	0	1	3	6	0	1	3	6
Particle size (nm)	206.1 ± 4.3	208.3 ± 4.4	210.9 ± 4.8	212.7 ± 5.1	206.1 ± 5.3	210.2 ± 5.5	212.4 ± 5.2	214.2 ± 5.8
Zeta potential (mV)	-18.0 ± 1.2	-.17.4 ± 1.0	-17.1 ± 1.1	-16.7 ± 1.12	-18.0 ± 1.2	-17.1 ± 1.01	-16.6 ± 1.15	-16.3 ± 1.2
PDI	0.20 ± 0.02	0.21 ± 0.03	0.22 ± 0.03	0.23 ± 0.025	0.20 ± 0.02	0.22 ± 0.01	0.24 ± 0.031	0.25 ± 0.037
Entrapment efficiency (%)	93.5 ± 1.3	91.9 ± 1.4	91.1 ± 1.5	89.11 ± 1.5	93.5 ± 1.3	90.2 ± 1.4	89.2 ± 1.7	88.17 ± 1.5

## Conclusion

 The *in-vitro* results demonstrated the efficiency of the nano cargoes in enhancing the dissolution profile, which directly correlates with improved bioavailability. The successful entrapment of the drug within the nano system, as evidenced by the results, indicates an improved elimination half-life. Additionally, the *in-vitro* release study suggested a reduced frequency, while stability studies indicated long-term stability, supporting the practicality of the designed system.

## Future prospective

 Conducting toxicity studies related to nanovesicles is essential. Additionally, attention must be given to *in-vivo* pharmacodynamic studies. Given the drug’s mechanism and its assumed inherent properties affecting the central and peripheral nervous systems, exploring an *in-vivo* model of epilepsy could also be beneficial.

## Competing Interests

 The authors report there are no competing interests to declare.

## Ethical Approval

 Quaid I Azam University, Islamabad, Pakistan (Ethical approval number: BEC-FBS-QAU2023-540).

## Supplementary Files


Supplementary file 1 contains Figures S1-S3 and Table S1.
