# Supplementary material for: Nano-Engineered Cargo for Optimizing Oral Absorption of Tizanidine Nanostructured Lipid Carriers
Source: Adv Pharm Bull. 2025 Oct 11;15(4):806–18. doi: 10.34172/apb.025.45650 (PMC12980190; doi:10.34172/apb.025.45650)
Supplement: Supplementary file 1 — contains Figures S1-S3 and Table S1. [file apb-15-806-s001.pdf]

Adv Pharm Bull. 2025;15(4):806-S1
doi: 10.34172/apb.025.45650
https://apb.tbzmed.ac.ir

Schematic illustration of how the preparation of NLCs ensued.

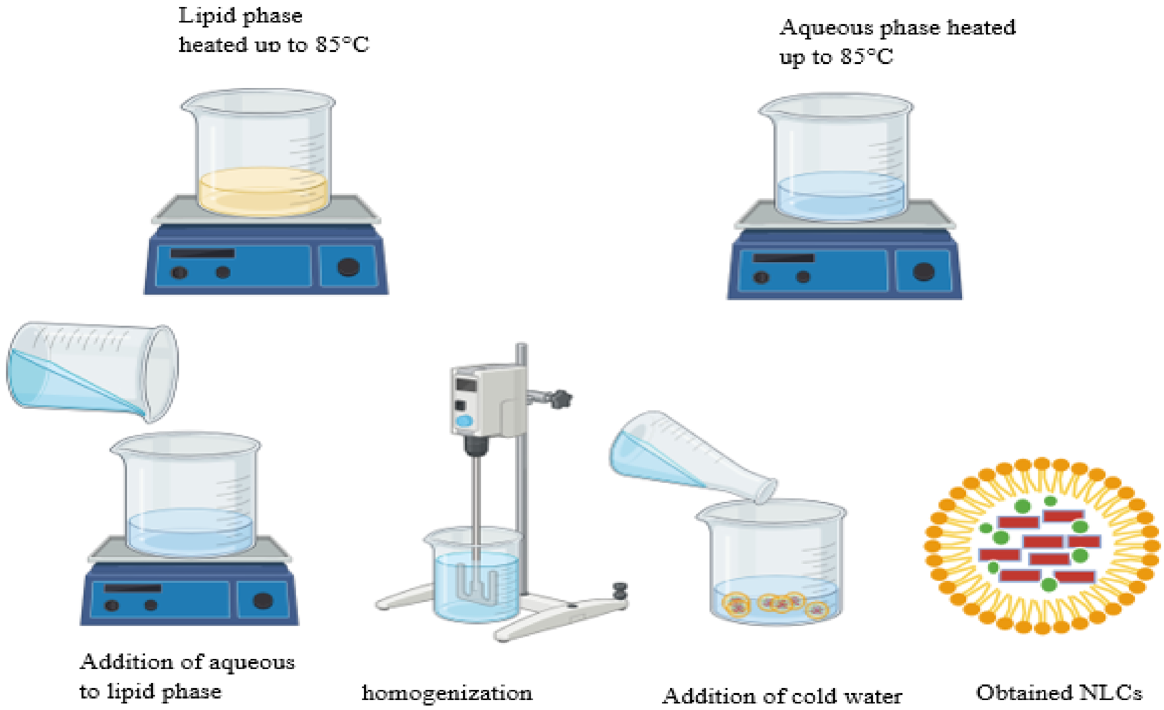

Figure S1: Preparative illustration of NLCs. Prepared via BioRender.

One factor plot for impact of variables on the Particle size

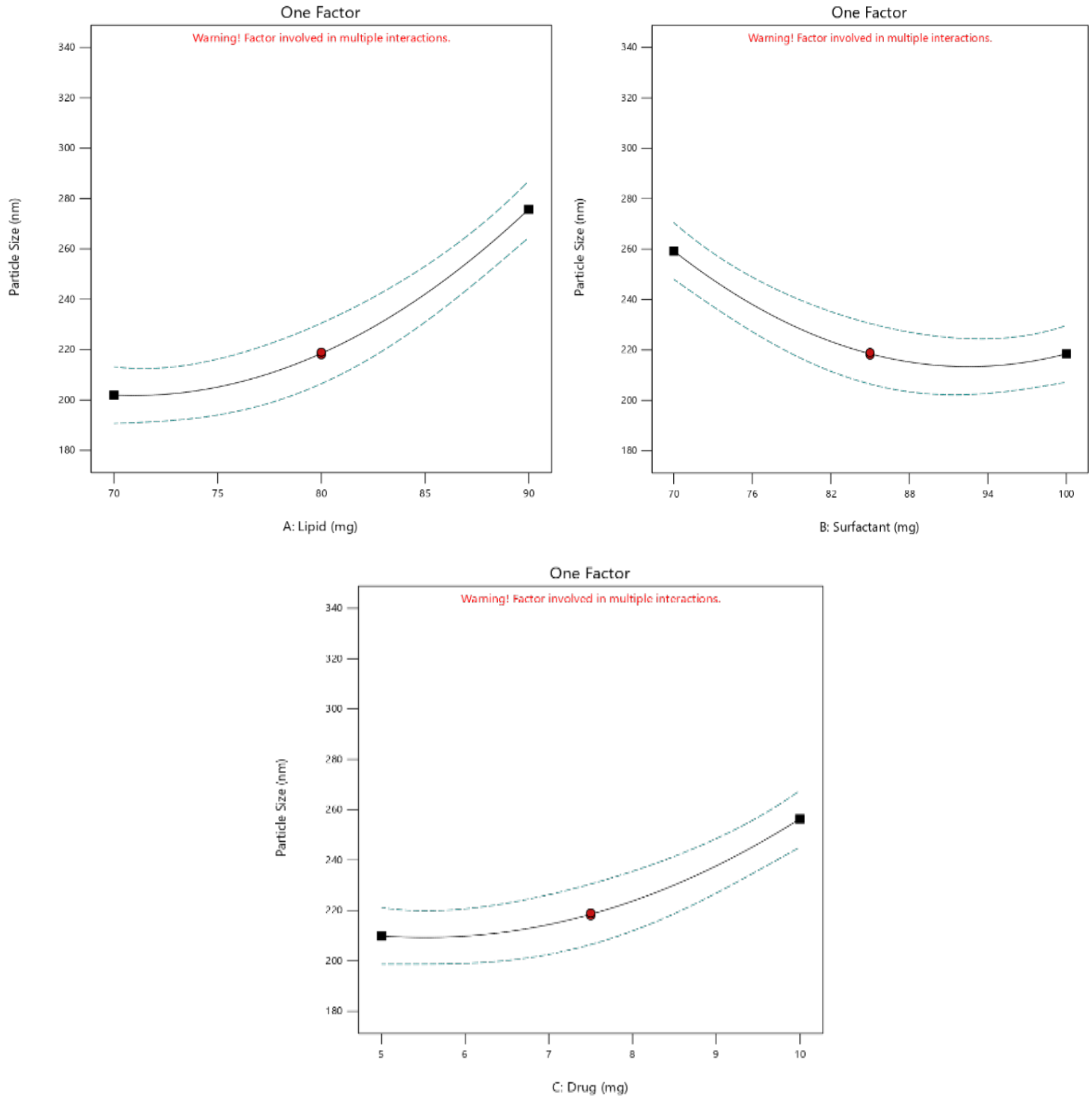

Figure S2. Impact of A. Lipids, B. Surfactant and C. Drug on Particle size (nm).

One factor plot for impact of variables on the Zeta Potential

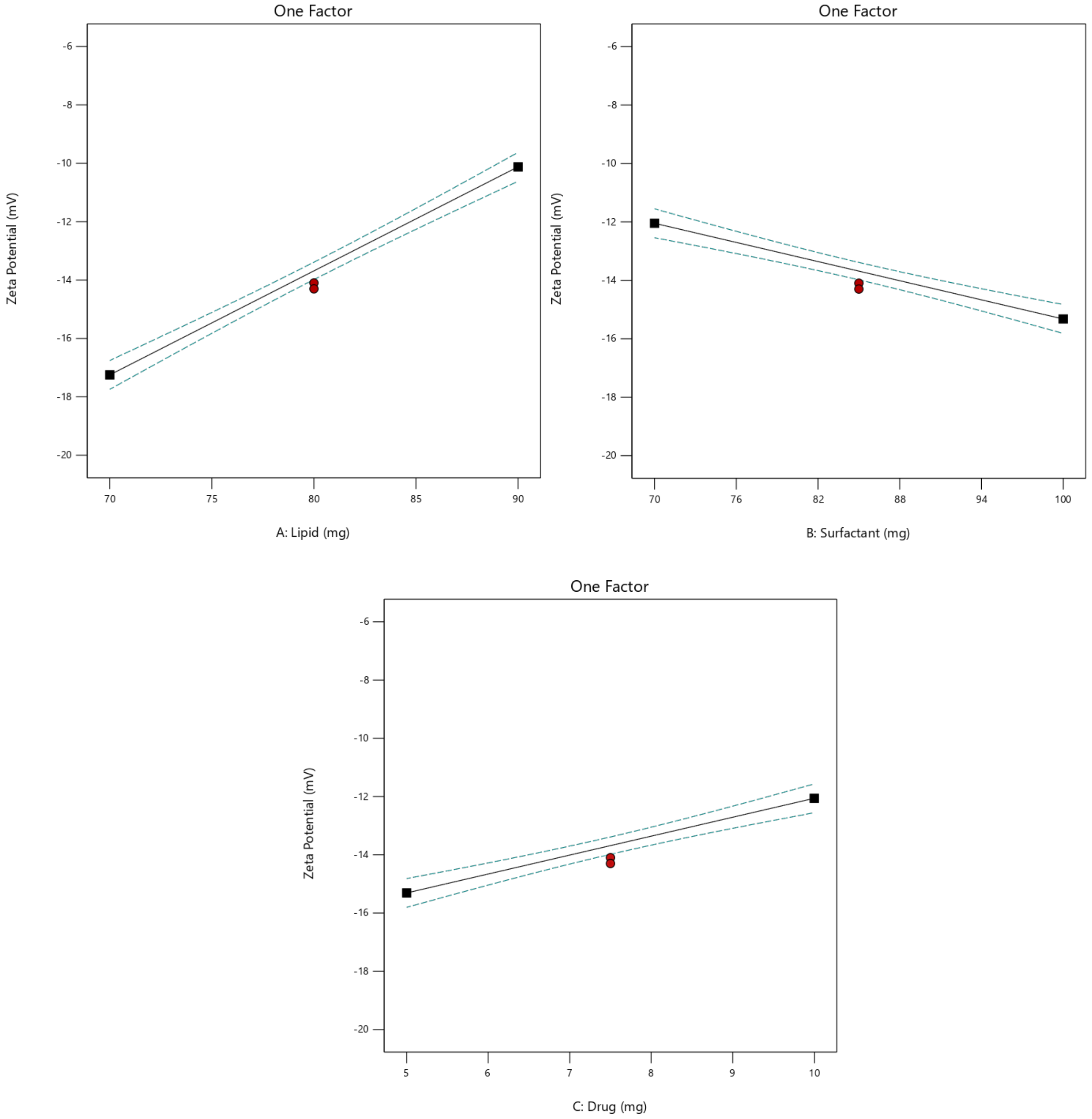

Figure S3. Impact of A. Lipids, B. Surfactant and C. Drug on the Zeta Potential (mV).

One factor plot for impact of variables on Entrapment efficiency

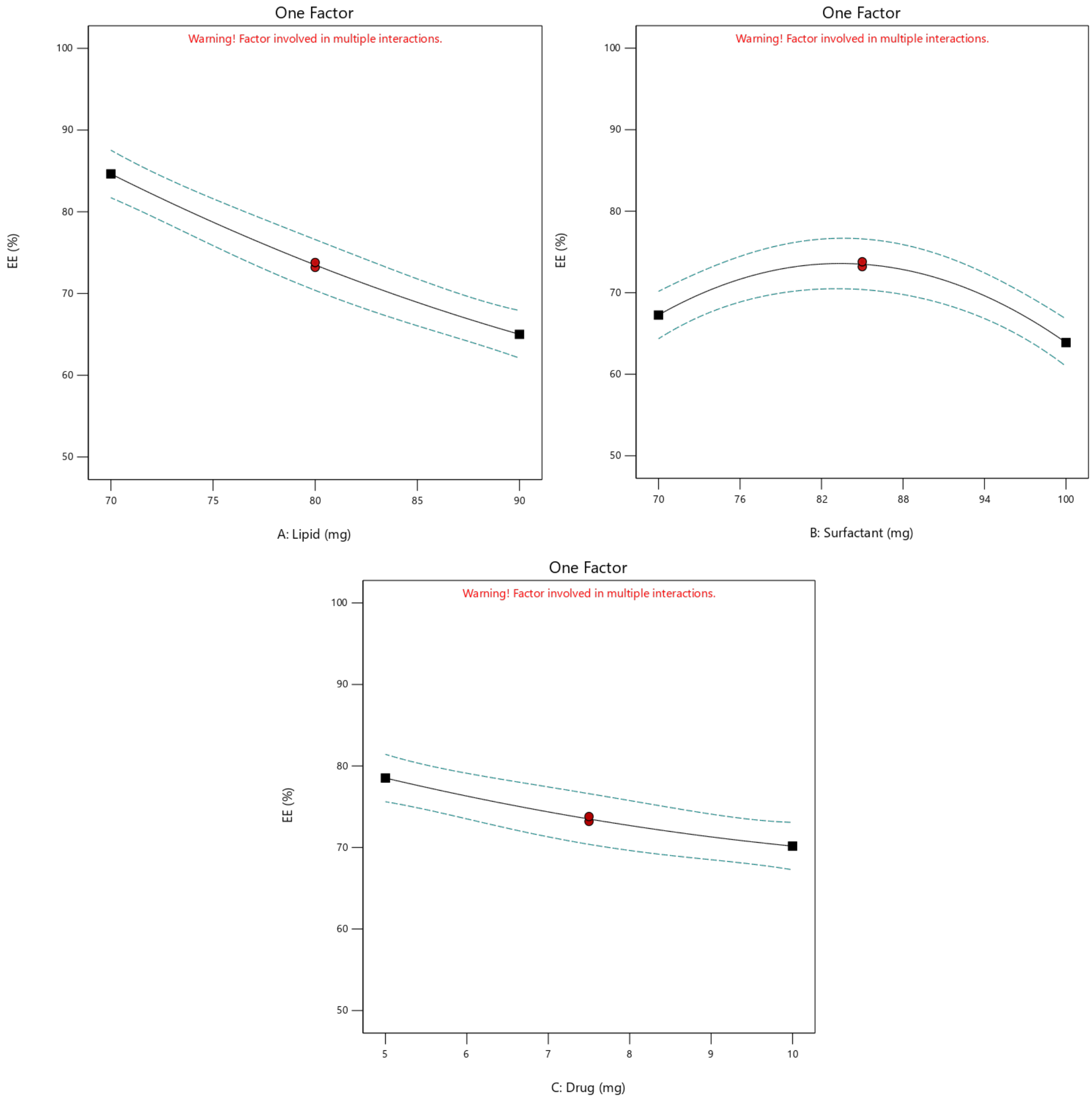

Figure S4. Impact of A. Lipid, B. Surfactant and C. Drug on Entrapment efficiency (%EE)

Kinetics models with R^2 value

Table S1. Kinetic Models with R2 values (TNZ NLCs)

**Models**	**Models R^2 values for TNZ NLCs**	
**Zero order**	0.6447	
**First order**	0.8859	
**Higuchi**	0.9892	
**Korsmeyer-Peppas**	R^2	0.9918
	Diffusion exponent (n)	0.462 (fickian release)
**Hixon-Crowell**	0.8252	
